# Discussion of costs and financial burden in clinical practice: A survey of medical oncologists in Australia

**DOI:** 10.1371/journal.pone.0273620

**Published:** 2022-10-21

**Authors:** Anupriya Agarwal, Deme J. Karikios, Martin R. Stockler, Rachael L. Morton

**Affiliations:** 1 NHMRC Clinical Trials Centre, The University of Sydney, Sydney, New South Wales, Australia; 2 Sydney Medical School, University of Sydney, Camperdown, New South Wales, Australia; 3 Nepean Cancer Centre, Nepean Hospital, Kingswood, New South Wales, Australia; 4 Concord Cancer Centre, Concord Repatriation General Hospital, Concord, New South Wales, Australia; 5 Chris O’Brien Lifehouse, Camperdown, New South Wales, Australia; Ashkelon Academic College, ISRAEL

## Abstract

**Background:**

A diagnosis of cancer is associated with significant physical, psychological and financial burden. Including costs of cancer is an important component of shared decision making. Doctors bear a responsibility towards educating patients about the financial aspects of care. Multiple organisations have advocated for price transparency and implementing Informed Financial Consent in the clinic. However, few studies have evaluated the perspectives of oncologists on the current state of this discussion.

**Aims:**

The aim of this study is to determine the views and perspectives of medical oncologists regarding communication of costs and financial burden in patients with cancer.

**Methods:**

We conducted a prospective cross-sectional online survey via REDCap. The survey was distributed to medical oncologists and advanced trainees currently registered with Medical Oncology Group of Australia (MOGA). Data was collected using the online survey comprising socio-demographic characteristics, discussion of costs and financial burden, and facilitators and barriers to these discussions.

**Results:**

547 members of MOGA were invited to participate in the study, and 106 of 547 MOGA members (19%) completed the survey. Most oncologists (66%) felt that it was their responsibility to discuss costs of care, however a majority of oncologists (59.3%) reported discussing costs with less than half of their patients. Only 25% of oncologists discussed financial concerns with more than half of their patients, and most oncologists were unfamiliar with cancer-related financial burden. Most Oncologists with greater clinical experience and those working in private practice were more likely to discuss costs with a majority of their patients.

**Conclusions:**

Certain characteristics of medical oncologists and their practices were associated with reported prevalence of discussing costs of care and financial burden with their patients. In the context of rising costs of cancer care, interventions targeting modifiable factors such as raising oncologist awareness of costs of care and financial burden, screening for financial toxicity and availability of costs information in an easily accessible manner, may help increase the frequency of patient-doctor discussions about costs of care, contributing to informed decision-making and higher-quality cancer care.

## Introduction

A diagnosis of cancer is associated with significant physical, psychological and financial burden. Costs of cancer care account for a substantial proportion of healthcare expenditure, and healthcare costs for cancer are higher than for other conditions [1]. Newer and more expensive therapies are being increasingly used, leading to improved survival, but at much higher cost. Discussions about costs of cancer care in patient-doctor consultations is an important component of shared decision making and more informed patient care. Discussions about costs can reduce the use of low-value treatments, and reduce costs overall [2], thereby enhancing value-based care.

Doctors bear a responsibility for educating patients about the financial aspects of cancer treatment. Multiple organisations [3], including the Australian Medical Association [4], have advocated for ‘fee- transparency’ as an essential component of patient-doctor communication. Additionally, the Cancer Council Australia has released a “Standard for Informed Financial Consent”, which requires oncologists to be transparent about costs of care [5]. However it is unclear to what extent oncology practices have implemented cost of care discussions; further information about how and when these discussions occur is needed [6, 7]. Studies of patient-provider conversations in other countries regarding costs and effects cancer treatment have shown low rates of cost discussions [8, 9], and inadequate clinician engagement in discussing the financial burden of cancer [10].

Few studies have evaluated the perspectives and attitudes of doctors with regards to discussions of costs of care or financial burden. We aimed to describe the frequency and nature of both cost of care and financial burden discussions in a clinical setting, and to determine the characteristics of medical oncologists and their practices that are associated with discussions about costs. We also sought to identify strategies and potentially modifiable factors to facilitate future discussions about costs of care and financial burden.

## Methods

We distributed an online survey to medical oncologists and medical oncology trainees practising in Australia that were listed on a professional association database (the Medical Oncology Association [MOGA]). MOGA is the national, professional organisation for medical oncologists in Australia. Participants were asked to recall discussions undertaken in the clinic over the last 12 months regarding the costs of cancer treatment, and the evaluation and management of financial burden. Clinical discussions included any conversations undertaken between clinicians and patients in outpatient clinics during a consultation. Socio-demographic characteristics of participants included: age, gender, qualifications, practice location, tumour streams, geographical region of practice, average number of patients seen per week, and practice type (public or private). The prevalence of discussions about costs of care was categorised as: with none of the patients, less than half of the patients, more than half of the patients, and all of the patients. These categories were dichotomised for analysis into, less than half of the patients, versus more than half or more of the patients. Discussions of financial burden were categorised in the same way.

The oncologist survey was developed by three medical oncologists (AA, DK, and MRS) and a health economist (RLM). Prior to wider distribution, the survey was piloted with two oncologists and two researchers to test clarity, content validity and face validity. The survey was delivered, distributed, and collected online using REDCap software.

### Statistical analysis

Descriptive statistics were reported to describe oncologist characteristics and the prevalence of discussions of costs of care, and financial burden. We quantified oncologist preferences for discussions of cost of care and financial burden using frequency distributions. To assess whether socio-demographic characteristics were associated with the frequency of discussions about costs of care (<50% vs >50%) and financial burden (<50% vs >50%), we built two multivariable logistic regression models. We reported unadjusted odds ratios (ORs), adjusted ORs, and 95% confidence intervals (CIs). First, we examined the univariate associations for each characteristic separately by examining the unadjusted odds ratios. We then built four multivariable regression models for each dependent variable in a stepwise approach. We included all characteristics of the oncologists, and then included only the characteristics that were significantly associated with the discussion of costs of care in the univariate analyses, and those that were relevant to the analysis including location of the oncology practice, average number of patients seen per week, and the type of practice. The final multivariable model included characteristics that were relevant to the discussion of out-of-pocket costs. Analyses were performed with SAS 9.4 and Graphpad PRISM 8 software.

This study was reported according to the STROBE checklist for observational studies [11].

## Results

547 members of MOGA were invited to participate in the study, and 106 of 547 MOGA members (19%) completed the survey. The majority of responders were qualified medical oncologists (83%), and approximately half were female (53%) (Table 1). 78% practiced in metropolitan regions and 64% were predominantly practising within the public healthcare system.

**Table 1:** Personal and professional characteristics of 106 responding oncologists.

Characteristic	n(%)
**Age**	
≤ 30	5 (5)
31–40	55 (52)
41–50	18 (17)
51–60	20 (19)
>61	7 (6)
No response	1
**Sex**	
Female	56 (53)
Male	50 (47)
**Qualifications**	
Advanced Trainee	18 (17)
FRACP < 5 yrs	35 (33)
FRACP > 5 yrs	53 (50)
**Practice Location**	
New South Wales	65 (61)
Queensland	8 (7)
Victoria	15 (14)
South Australia	7 (6)
Western Australia	6 (5)
Tasmania	1
ACT	3 (2)
Northern Territory	1
**Tumour stream**	
CNS	25 (23)
Breast	57 (54)
GI	63 (59)
GU	49 (46)
Gynae	32 (30)
H&N	25 (23)
Lung	61 (57)
Melanoma	32 (30)
Sarcoma	13 (12)
Neuroendocrine	20 (19)
Other:	4 (3)
Research	2
Early phase clinical trials	1
Geriatric oncology	1
**Type of practice**	
Metropolitan	83 (78)
Regional	19 (18)
Remote/Rural	3 (2)
**Average number of patients seen/week**	
Less than 10 pts	14 (13)
Between 11 – 30pts	32 (30)
Between 31–50 pts	31 (29)
More than 50 pts	18 (17)

### Discussions of costs of care

In response to how often oncologists discussed costs of cancer care with patients in the past 12 months, 3.7% reported never discussing costs; 55.6% reported discussing costs with less than half their patients; 33.9% discussed costs with more than half their patients and 5.6% discussed costs with all their patients.

**Fig 1:**
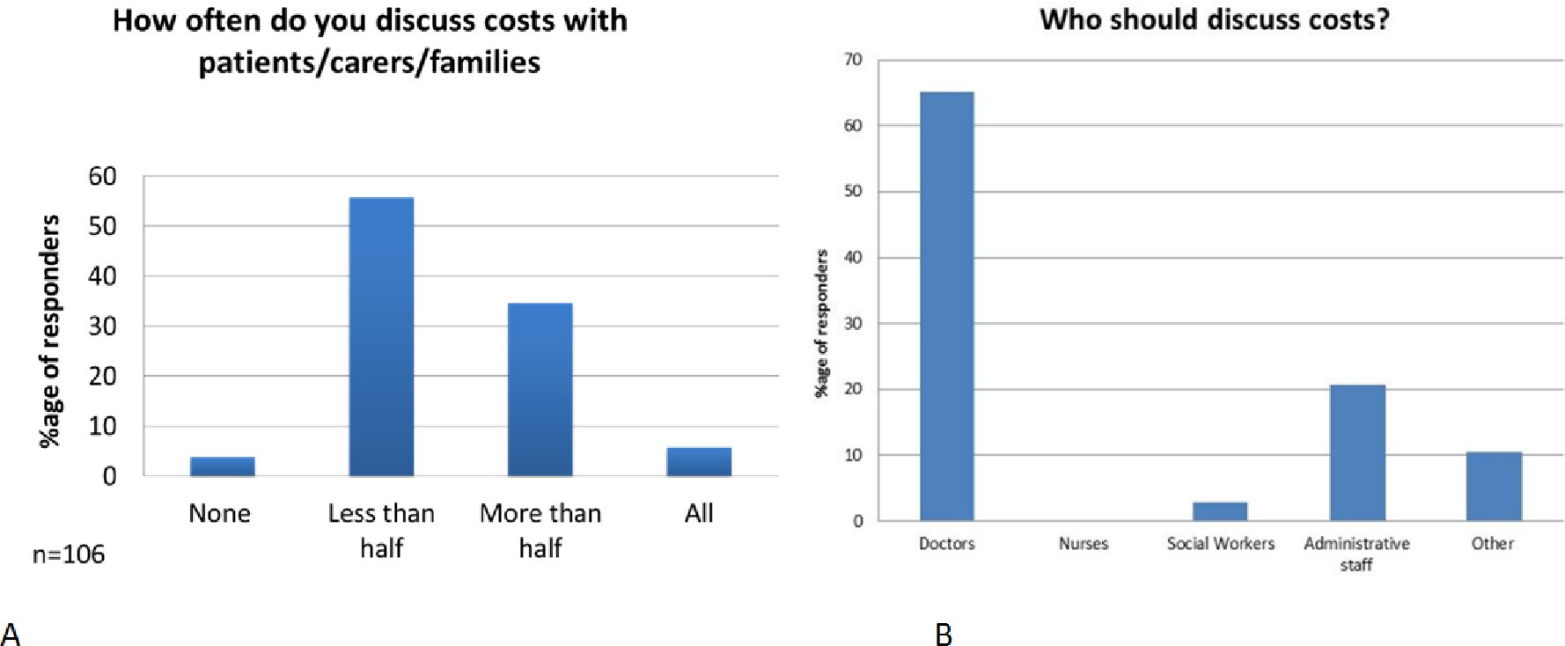
Discussions of costs in clinical practice. A: Reported frequency of discussions about costs in the past 12 months by oncologists. B: Clinic personnel reported as being responsible for discussion of costs.

Most oncologists (66%) felt that it was their responsibility to discuss costs of care, rather than that of other healthcare professionals in the clinic (Fig 1). Most (51%) felt ‘somewhat confident’ to conduct these cost of care discussions; whereas 26% felt very confident, 20% not so confident, 3% not at all confident, and 2% extremely confident.

**Fig 2:**
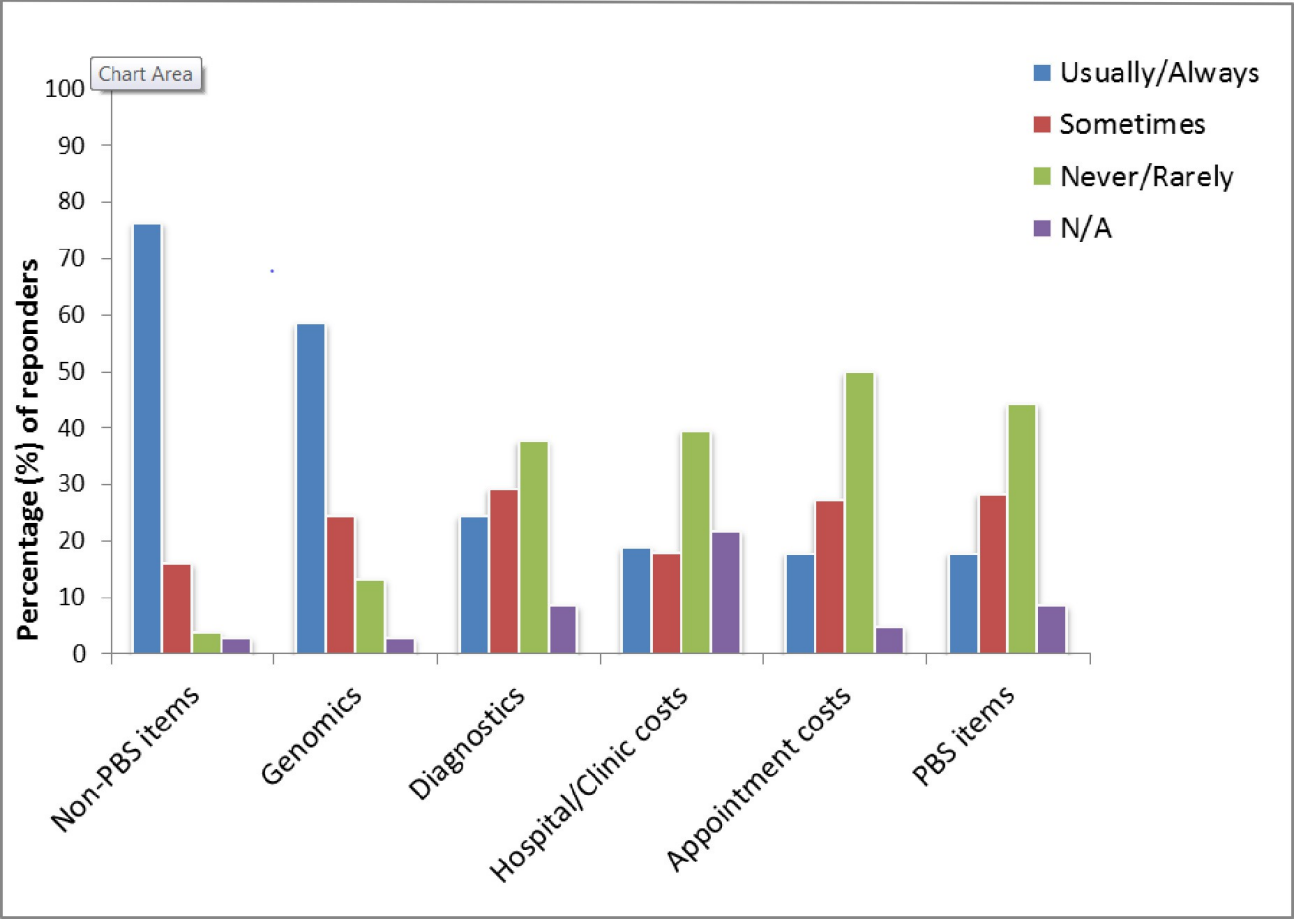
Reported frequency of discussions in the last 12 months about specified out-of-pocket costs. PBS–Pharmaceutical Benefits Scheme (medicines subsidised by the government). Non-PBS–Not on the Pharmaceutical Benefits Scheme (medicines not subsidised by the government).

The items doctors were most likely to discuss with patients were treatments not listed for reimbursement on the Pharmaceutical Benefits Scheme (PBS), and genomic tests not subsidised by Medicare (Fig 2). Oncologists were most likely to either never discuss or rarely discuss the cost of items that were listed on the PBS, diagnostic tests (pathology, imaging), clinic consultation fees, or costs of appointments with other healthcare professionals.

**Fig 3:**
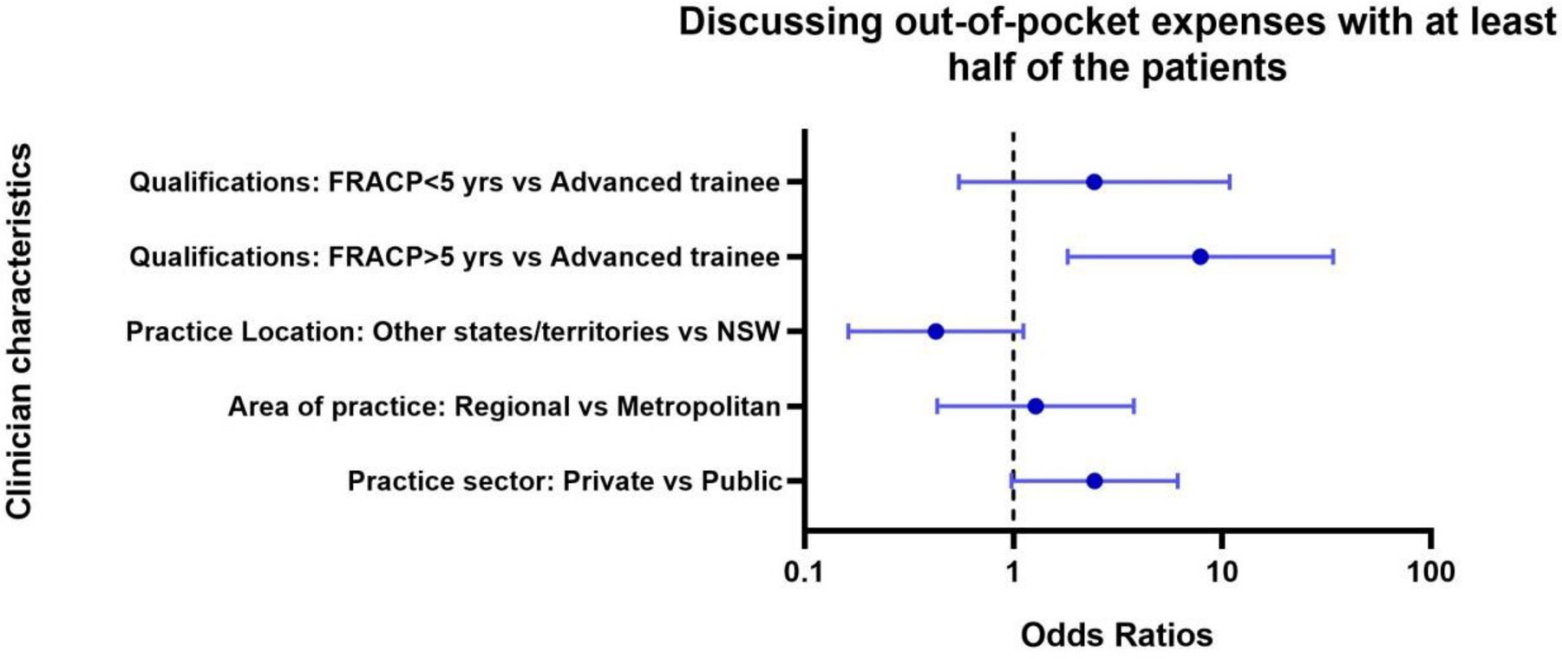
Characteristics associated with reported frequency of discussions of out-of-pocket costs with patients. *FRACP = Fellow of Royal Australasian College of Physicians.

The following clinical characteristics were associated with discussing costs of care with more than 50% of patients: oncologists with greater clinical experience, and those working in private practice. Oncologists with greater than 5 years of practice since attaining specialist qualifications were more likely to discuss costs of care compared with their junior counterparts OR 7.8 (95%CI 1.8 to 34, p = 0.01) (Fig 3). Oncologists who worked in private practices were more likely to discuss costs of care (OR 2.4, 95% CI 0.98 to 6.1) than those who only worked in the public health system.

**Fig 4:**
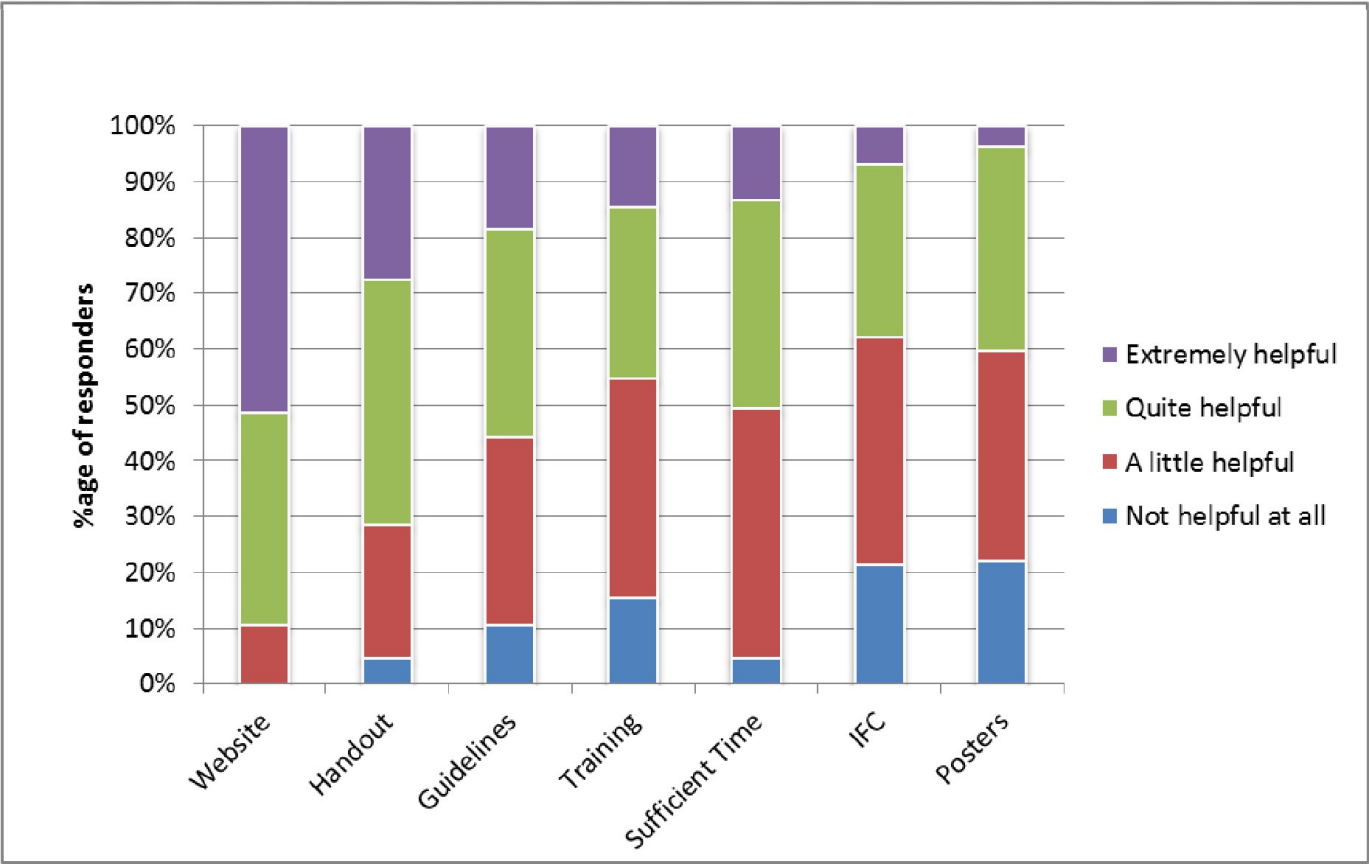
Strategies to facilitate discussion of out-of-pocket (OOP) costs in the clinic. *IFC = Standard for Informed Financial Consent.

Oncologists were asked to rate the influence of current and potential strategies that could facilitate discussions about costs of care in their clinics. Facilitators that were rated by oncologists as extremely helpful included: availability of cost information on a website (51% of oncologists), and handouts for patients with information about costs (28%) (Fig 4). Strategies less likely to be reported helpful included: guidelines for oncologists on effective costs communication (18%), clinician training on cost of care communication (14%), and having sufficient time to discuss costs (13%). Strategies that were rated not helpful were: posters reminding patients to ask about costs (22%), and the requirement to discuss costs as a component of informed financial consent (21%).

**Fig 5:**
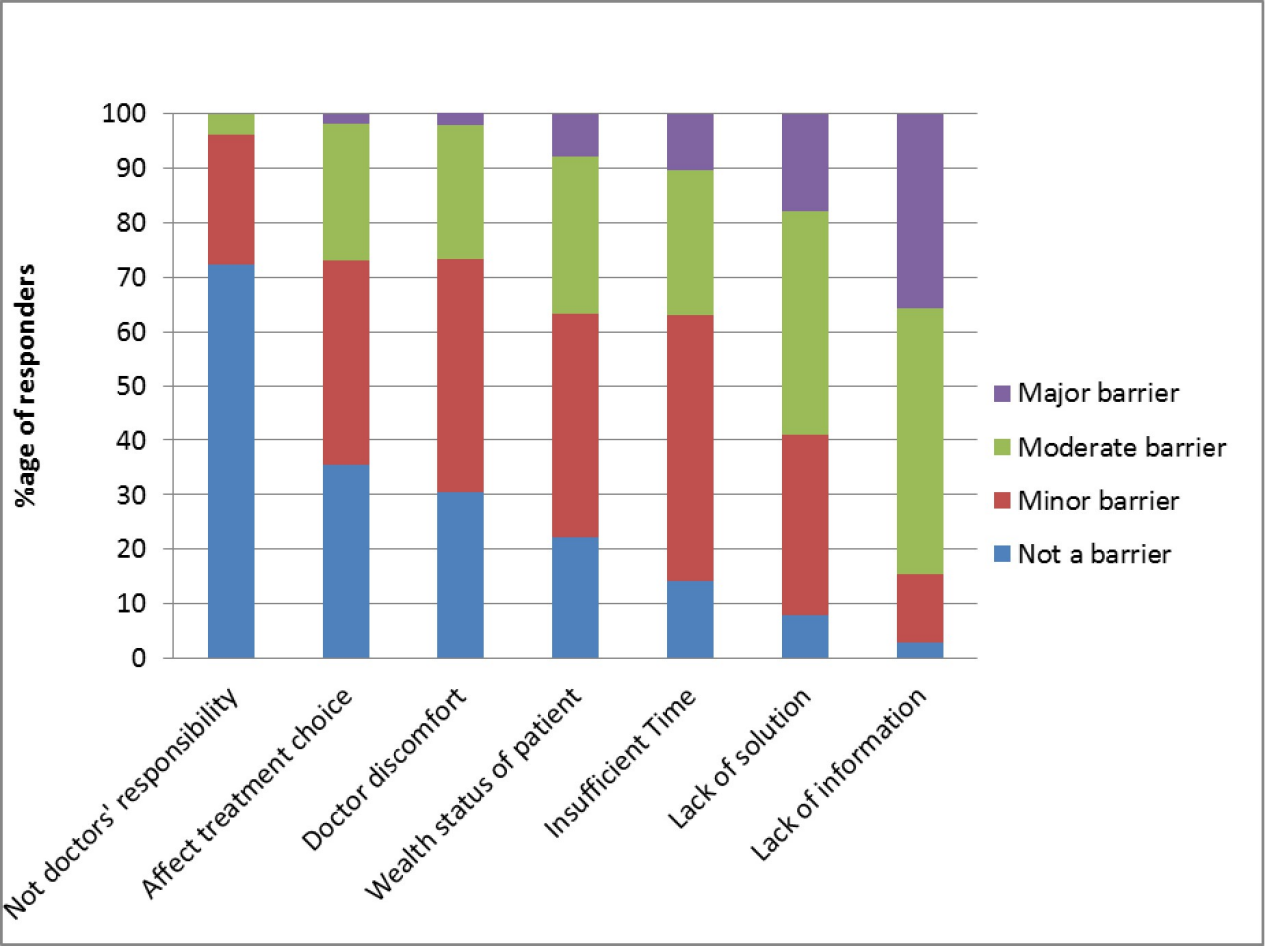
Current and potential barriers to discussion of costs in clinics by oncologists.

Clinicians were also asked to rate the influence of potential barriers to discussions of costs of care during a clinical consultation. Oncologists reported lack of available information about costs (36%), and limited solutions for cost concerns (18%) as major barriers for discussing costs of care with patients (Fig 5). Clinicians were asked whether they felt it was not their responsibility to discuss costs with patients, and only a quarter of clinicians (28%) felt that a lack of doctors’ responsibility of costs discussion was a barrier to fee transparency. 36% of clinicians reported that ‘costs of care potentially influencing treatment choices’ was not a barrier to discussing costs.

### Discussions of financial burden

In response to the question about how frequently oncologists discussed financial concerns with patients and/or their families, 4% reported never having discussed it, 71% that they had discussed it with less than half their patients, 22% with more than half their patients, and 3% with all their patients. Approximately half the oncologists (48%) reported that discussion of financial burden was generally initiated by patients, one-third (32%) reported the topic was usually initiated by doctors, and one-sixth (17%) that it was generally initiated by family members or carers, 3% that they were initiated by a member of the allied health team.

**Fig 6:**
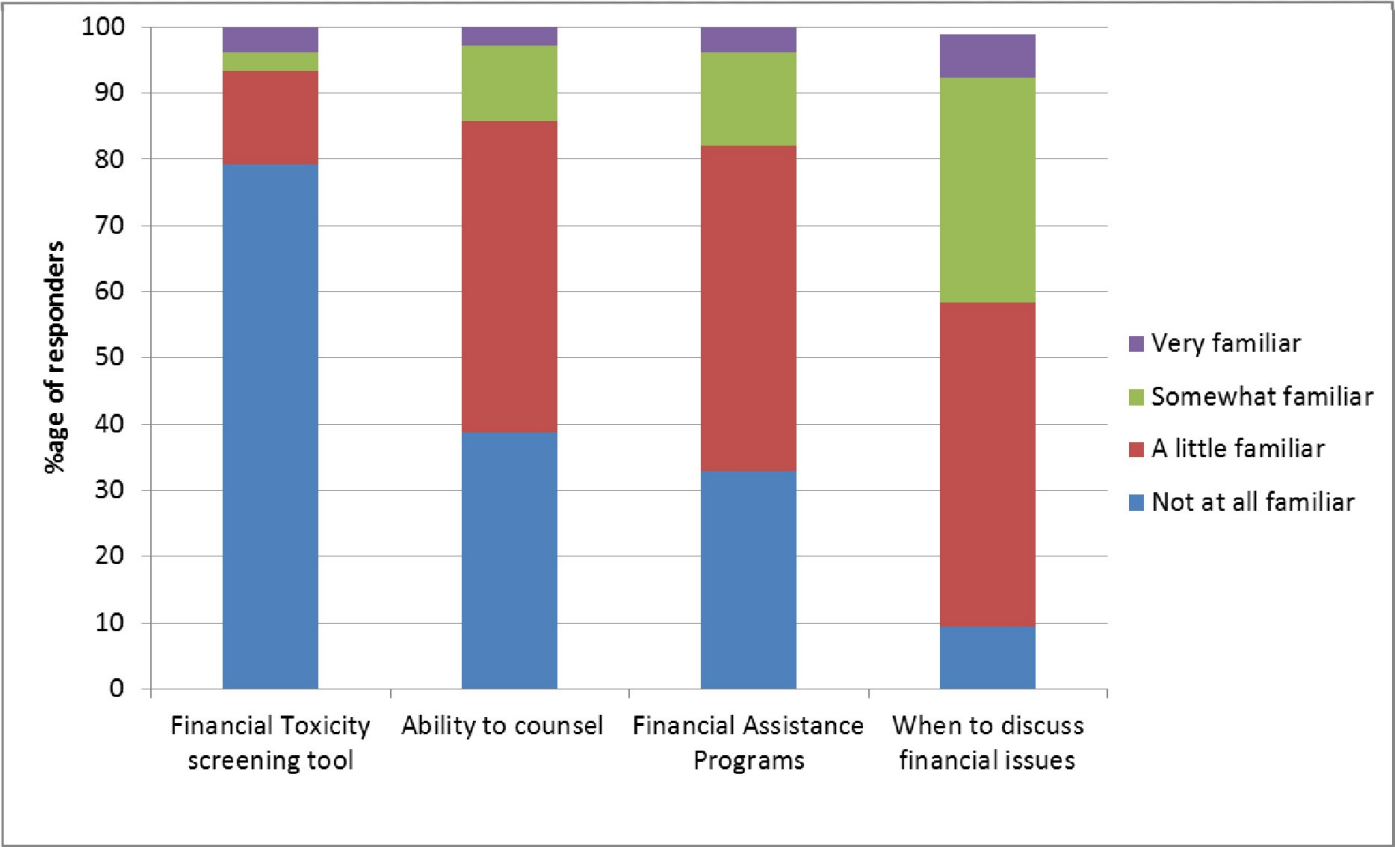
Oncologists’ familiarity with aspects of financial burden.

Respondents reported limited knowledge of cancer-related financial burden, with most (93%) reporting little or no familiarity with a financial toxicity screening tool e.g. COST-FACIT [12, 13]; little or no ability to provide counselling to patients suffering financial burden (86%); little knowledge of financial assistance programs available to patients (82%); and little knowledge about the ideal time to screen for financial issues in consultations (58%) (Fig 6).

Despite this, approximately half of the respondents 56 of 106, (53%) had referred patients to financial assistance programs, financial resources, or counselling to assist with financial burden. A smaller proportion 30 of 106, (28%) had not made a referral themselves, but h relied on a different healthcare professional in their service to make appropriate referrals. One in seven respondents (14%) were not aware of any pertinent financial assistance programs or counselling, and 5 clinicians reported that they had no programs in their service to offer.

**Fig 7:**
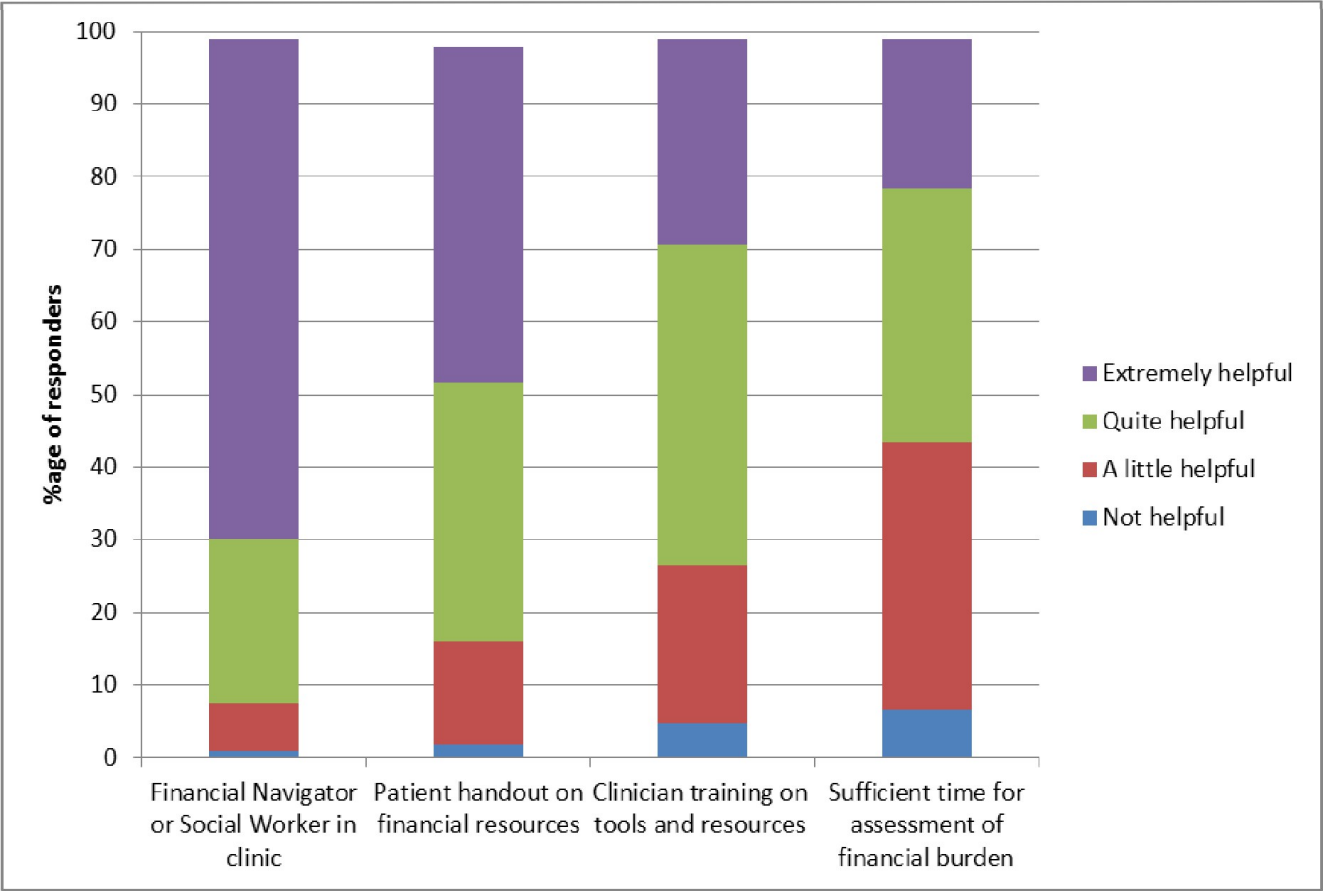
Oncologists’ views on tools to assist in management of financial burden in the clinic.

Clinicians were asked to rate the helpfulness of current and potential strategies that could facilitate discussion of financial burden in the clinic. Most oncologists, 73 of 106, (69%) reported that the availability of a financial navigator or social worker in the clinic would be extremely helpful (Fig 7). Other strategies that were reported to be extremely helpful were, a patient handout on available financial resources reported by 49 of 106 (46%), clinician training about tools to assess financial burden and resources available reported by 30 of 106 (28%), and sufficient time to perform screening and counselling for financial burden reported by 22 of 106 (21%).

Tools and resources reported to be most useful in assisting with communicating costs of care and financial burden were, a financial navigator in the clinic to discuss costs and resources with patients directly reported by 88 of 106 (83%), online healthcare cost information to assist clinicians in estimating expected costs of care reported by 72 of 106 (68%) and, online education modules on financial burden assessment and management reported by 42 of 106(40%). Resources that were less frequently found to be useful were an implementation pack for informed financial consent with financial burden screening tools and resources by 36 of 106 (33.9%), electronic medical record (EMR) flags or posters to prompt discussions of costs of care or financial burden with patients reported by 32 of 106 (30.2%), and interactive communication workshops on financial burden reported by 30 of 106(28.3%).

## Discussion

This is the first survey of Australian medical oncologists regarding their practices and perspectives on discussions of costs of care and financial burden in patients with cancer. The study identified that oncologists thought it was their responsibility to discuss costs of care and financial burden with their patients. Oncologists who were qualified for more than five years, and those who worked in private practice, were more likely to report discussing the costs of cancer care with a majority of their patients than those who were early in their career (less than 5 years since graduation) or worked predominantly in public healthcare settings. We did not identify any oncologist characteristics associated with discussing financial burden. Approximately two-thirds of oncologists felt it was their responsibility to discuss of care, particularly out-of-pocket costs however, more than half of respondents reported discussing costs with less than half of their patients.

In the past decade, there has been increased awareness of the rising costs of cancer care and financial burden for people affected by cancer. This has led numerous medical organisations in Australia and overseas to introduce standards for informed financial consent [3–5]. In addition, current clinical practice guidelines recommend increased clinician awareness of cost variability and high total costs of treatment. Guidelines recommend that clinicians explore concerns about costs of care and address financial worries, especially in groups at high risk for developing financial toxicity [14]. As personalised treatments for cancer become more available, oncologists will increasingly refer patients for genomic testing and recommend expensive, molecularly-targeted treatments. Improving patient-doctor discussions about expected costs of care will be critical to ensure patients can make informed decisions and plan for treatment expenses. Cost-conversations can help address costs of care by directing patients to available resources to reduce the incidence and severity of financial burden [2, 15].

This study has limitations inherent to all surveys. The response rate was low but consistent with other studies that recruit through a professional association’s mailing list, and as with all surveys, there may have been differences between responders and non-responders. A majority of responders were from a single state (New South Wales), and although this is the most populous state in Australia, it is possible that there was greater awareness of cancer costs and financial burden due to the Cancer Council Financial Assistance programs [16].

This study highlighted possible facilitators and barriers to discussing costs and financial burden from the oncologists’ perspective. We identified characteristics of oncologists and their practices that were associated with increased prevalence of cost of care discussions, including greater number of years since qualification and work in a private practice. A greater number of years in practice and higher patient volumes are known to be associated with aspects of cost-consciousness and awareness of patient out-of-pocket costs among clinicians [17]. Oncologists in private practice reported discussing costs with a higher proportion of their patients, potentially because the cost of care in private practice is higher than for those treated in a public hospital, including the costs of private health insurance, gap payments, and other out-of-pocket costs [18]. The findings of this study can assist in guiding effective communication about the costs of care and improve provider-level interventions to address financial burden for people with cancer. A majority of clinicians reported three significant facilitators to discussion of costs of care (i)availability of cost information on a website, (ii) out-of-pocket cost estimates outlined in a patient handout, and (iii) clinician training on effective communication about costs of care. These facilitators can be easily implemented into clinical practice with development of a website and/or handout with expected costs estimates. Clinician training and awareness of costs of care and financial burden can be delivered in the form of a module for continuing medical education and professional development.

The findings of this study have significant implications for the future training and practice of medical oncologists regarding the implementation of cost transparency and assessment of financial burden. Despite the introduction of a standard for informed financial consent in medical oncology in Australia [5], clinicians were less likely to report implementation of this consent to facilitate a cost of care discussion. This is likely due to the lack of easily available information about costs of care for oncologists to discuss with patients, and differences in costs between practice types, locations, and tumour types [18–20].

In addition, communication workshops and education modules outlining effective discussion of costs and financial burden can be implemented at different levels of doctor training from undergraduate degrees university to post-graduate qualifications. Clinicians feel a responsibility to discuss costs of care, however, they may face moral and ethical dilemmas regarding recommendation of unfunded or expensive treatments to patients with pre-existing financial burden [21]. Clinician guideline on cost conversations and further qualitative research into supportive measures is needed to ensure effective shared-decision making.

In conclusion, we found characteristics of medical oncologists and their practices were associated with reported prevalence of discussing costs of care and financial burden with their patients. In the context of rising costs of cancer care, interventions targeting modifiable factors such as raising oncologist awareness of costs of care and financial burden, screening for financial toxicity and availability of costs information in an easily accessible manner, may help increase the frequency of patient-doctor discussions about costs of care, contributing to informed decision-making and higher-quality cancer care.

## Supporting information

S1 Data (CSV)
